# Pairing Cholinergic Enhancement with Perceptual Training Promotes Recovery of Age-Related Changes in Rat Primary Auditory Cortex

**DOI:** 10.1155/2016/1801979

**Published:** 2015-12-29

**Authors:** Patrice Voss, Maryse Thomas, You Chien Chou, José Miguel Cisneros-Franco, Lydia Ouellet, Etienne de Villers-Sidani

**Affiliations:** Department of Neurology and Neurosurgery, Montreal Neurological Institute, McGill University, Montreal, QC, Canada H3A 2B4

## Abstract

We used the rat primary auditory cortex (A1) as a model to probe the effects of cholinergic enhancement on perceptual learning and auditory processing mechanisms in both young and old animals. Rats learned to perform a two-tone frequency discrimination task over the course of two weeks, combined with either the administration of a cholinesterase inhibitor or saline. We found that while both age groups learned the task more quickly through cholinergic enhancement, the young did so by improving target detection, whereas the old did so by inhibiting erroneous responses to nontarget stimuli. We also found that cholinergic enhancement led to marked functional and structural changes within A1 in both young and old rats. Importantly, we found that several functional changes observed in the old rats, particularly those relating to the processing and inhibition of nontargets, produced cortical processing features that resembled those of young untrained rats more so than those of older adult rats. Overall, these findings demonstrate that combining auditory training with neuromodulation of the cholinergic system can restore many of the auditory cortical functional deficits observed as a result of normal aging and add to the growing body of evidence demonstrating that many age-related perceptual and neuroplastic changes are reversible.

## 1. Introduction

Perceptual learning involves relatively long-lasting changes to organism's perceptual systems that improve its ability to respond to its environment [1]. In an experimental setting, this generally translates to an improvement in performance on a perceptual task with training. One of the defining characteristics of perceptual learning is its specificity to the physical parameters of the stimuli used for training [2]. For instance, when learning to discriminate between different directions of motion, the improvement does not fully generalize to other directions of motion the subjects were not trained on [3]. Similarly, listeners who are trained to discriminate between different pitch sensation-inducing amplitude modulated noises showed no more improvement than untrained listeners at discrimination between pure tones or noise bursts with different amplitude modulation rates [4]. Perceptual learning also leads to marked cortical plasticity within sensory cortex showing a similar level of specificity (see [5]). One well-known example in the animal auditory system is the finding of spatially enlarged frequency representations that are specific to tone frequencies that owl monkeys were trained to discriminate [6]. Similarly, within the visual system, orientation discrimination training has been shown to produce sharper tuning curves in V1 neurons, but again only for the trained orientations [7].

A growing body of evidence has suggested that perceptual learning and its associated cortical plasticity can also be boosted by neuromodulation. The cholinergic system in particular, which uses acetylcholine (ACh) as a neurotransmitter, has been shown to be a potent neuromodulatory system that plays critical roles in cortical plasticity, attention, and learning [8]. Indeed, neurochemically boosting cholinergic transmission [9–11] and stimulating the basal forebrain from which the cholinergic neurons project to the cortex [12–14] have both been shown to have a significant effect on both learning and the cortical processing of stimuli. Consequently, the activation of the cholinergic system during perceptual training leads to a long-lasting shaping of cortical circuits that forms the basis of learning.

The cholinergic system is also known to undergo significant changes with aging. For instance, the basal cholinergic cells tend to degenerate with advancing age [15, 16], which in turn has been shown to affect afferent cortical projections [17, 18]. These age-related changes have often been thought to contribute to the attentional and cognitive deficits observed during aging [19, 20]. Consequently, it has been hypothesized that boosting brain function through cholinergic enhancement during rehabilitation paradigms might help individuals with cognitive or sensory deficits related to aging with the hope of not only recovering sensory abilities, but also promoting brain plasticity. Indeed, the pharmacological potentiation of cholinergic neurotransmission has been shown to improve performance on cognitive tasks in the elderly [21–23] and chronic treatment with drugs that enhance cholinergic function has been used to ameliorate cognitive dysfunction [24, 25].

What remains particularly unclear at this point is whether this potentiation effect is modulated by age, and if so in what manner? While it is already established that young and old individuals learn sensory tasks at different rates [26–29], it remains to be determined whether cholinergic potentiation will provide similar behavioral gains for both age groups. Furthermore, it is equally unclear whether enhancing cholinergic transmission in both age groups will differentially affect cortical sensory representations. Consequently, the purpose of the present study was to investigate the potentially differential effect of a cholinesterase inhibitor (rivastigmine tartrate) on both brain function and behavior in young and old adult rats. Cholinesterase inhibitors are a class of drugs that raise the level of ACh in the brain by inhibiting the activity of the cholinesterase enzyme that metabolizes ACh [30], thus providing a potent cholinergic enhancement by increasing both the level and duration of the neurotransmitter action. Here, we used the rat primary auditory cortex (A1) as it has repeatedly proven to be an excellent model to study brain plasticity where perceptual learning is often reflected in the training-specific refinement of auditory cortical representations in both young and aging brains [29, 31, 32]. We hypothesize that while both cholinergic-boosted age groups compared to controls treated with saline placebo will show increased learning rates when performing a two-tone discrimination task, the improvement might be greater in the older rats given the greater room for improvement. Similarly, we expect that the neural representations of auditory cortical neurons in the older rats will show more plastic training-induced changes and that these same neurons will display young-adult functional properties to a greater extent following training (see [29]).

## 2. Methods

All experimental procedures used in this study were approved by the Montreal Neurological Institute Animal Care Committee and follow the guidelines of the Canadian Council on Animal Care. Eighteen old (O: 24–30 months) and nineteen young (Y: 12–14 months) Brown-Norway rats were used for this study. Within each age group, rats were divided into one of three groups: untrained (Y-UT (*n* = 8) and O-UT (*n* = 8)), trained while being orally given rivastigmine tartrate (Y-TR (*n* = 6) and O-TR (*n* = 4)), and trained in combination with saline administration (Y-TS (*n* = 4) and O-TS (*n* = 5)). All rats had unrestrained access to water and were housed in an environment with a 12-hour light/dark cycle. Those that underwent behavioral training were lightly food deprived.

### 2.1. Training Procedure

The rats' behavior was shaped in three phases. During the first phase, rats were trained to make a nose poke response to obtain a food reward. During the second phase, rats were trained to make a nose poke only after presentation of an auditory stimulus. During the third phase, the actual training program, rats were trained to make a nose poke only for the target stimulus (a 5 kHz pure tone) and not for a foil nontarget stimulus (10 kHz pure tone). The tones were presented at 60 dB SPL, stimulus presentation was randomized, and the probability of a target stimulus presentation was set at 20%. Training was performed in an acoustically transparent operant training chamber (60 × 45 × 35 cm, length × width × height) contained within a sound-attenuated chamber. Sound presentation and response recording were performed using the OpenEx software and RZ6 auditory processing hardware from TDT (Tucker-Davis Technology, Alachua, FL) and delivered in a free field manner through a calibrated loudspeaker.

The intertrial interval was selected at random from a range of 4 to 6 s. A rat's behavioral state at any point in time was classified as either “go” (producing a nose poke behavior) or “no-go.” For a given trial, the rat could elicit one of four reinforcements produced by the combinations of responses (go or no-go) and stimulus properties (target or nontarget). Go responses within 5 s of a target were scored as a* hit*; a failure to respond within this time window was scored as a* miss*; a go response within 5 s of a nontarget stimulus was scored as a* false positive*; the absence of a response was scored as a* withhold*. A hit triggered the delivery of a food pellet. A miss or false positive initiated a 5 s “time-out” period during which time the house lights were turned off and no stimuli were presented. A withhold did not produce a reward or a time-out. Psychometric functions and stimulus target recognition indexes (*d*-prime) were calculated for each training session by plotting the percentage of go responses as a function of the total number of target stimuli (i.e., hit ratio) and the percentage of false positives as a function of the total number of foils (i.e., false positive ratio). Learning curves were reconstructed by plotting the* d*-prime measure reached over successive days of training.

Thirty minutes prior to each training session, rats were orally given either a 0.2 mg/kg dosage of the cholinesterase inhibitor rivastigmine tartrate (Y-TR and O-TR groups) or an equal quantity of saline (Y-TS and O-TS groups). The dosage was calculated as a function of the recommended daily dose in humans. The specific timing of the administration of the drug was selected so that the entire training session was completed by the elimination half-life time of the drug (1.5 hrs). The duration of each behavioral training session lasted one hour and all animals were trained five days per week. All behaviorally trained animals had completed between 9 and 12 training sessions (phase 3) prior to undergoing electrophysiological recordings. The average number of training sessions did not differ between groups (Y-TR: 11.5 ± 0.55, Y-TS: 11.75 ± 0.5; O-TR: 11.75 ± 0.5, O-TS: 11 ± 1.41; *F* = 0.789, *p* = 0.518).

### 2.2. Electrophysiological Recordings

For A1 mapping, the rats were premedicated with dexamethasone (0.2 mg/kg) to minimize brain edema. They were then anesthetized with ketamine/xylazine/acepromazine (65/13/1.5 mg/kg, i.p.) followed by a continuous delivery of isoflurane 1% in oxygen delivered via tracheostomy intubation (after a tracheotomy was performed) and mechanical ventilation. Vital signs were continuously recorded using a MouseOx device (Starr Life Sciences, Holliston, Massachusetts). Body temperature was monitored with a rectal probe and maintained at approximately 37°C with a homeothermic blanket system. The absence of reflexes and stable heart rate indicated a deep anesthesia.

The rats were placed in a custom designed head holder, holding the rat by the orbits, leaving the ears unobstructed. The cisterna magnum was drained of cerebrospinal fluid to further minimize cerebral edema. The right temporalis muscle was reflected, auditory cortex was exposed via craniotomy, and the dura was resected. The cortex was maintained under a thin layer of silicone oil to prevent desiccation. Cortical responses were recorded with 64-channel tungsten microelectrode arrays (TDT, Alachua, FL). The microelectrode array was positioned above auditory cortex and was lowered orthogonally into the cortex to a depth of approximately 500–650 *μ*m (layers 4/5), where vigorous stimulus-driven responses were obtained. Penetration sites were chosen to avoid blood vessels.

The extracellular neural action potentials were amplified, filtered (0.3–5 kHz), and monitored on-line. A combination of multi- and single-unit activities was used to reconstruct characteristic frequency maps. For response bandwidths 20 dB above threshold (BW20), only single unit data was used. Spike sorting was performed with an automated algorithm using principal component analysis (OpenSorter; Tucker-Davis Technology, Alachua, FL). Acoustic stimuli were generated using TDT System III (Tucker-Davis Technology, Alachua, FL) and delivered in a free field manner to the right ear through a calibrated speaker (TDT). A software package (OpenEx; Tucker-Davis Technology, Alachua, FL) was used to generate acoustic stimuli, monitor cortical response properties on-line, and store data for off-line analysis. The evoked spikes of a single neuron or a small cluster of neurons were collected at each site in the hemisphere (left) contralateral to the stimulated ear. Frequency-intensity receptive fields (RF) were reconstructed by presenting pure tones of 63 frequencies (1–48 kHz; 0.1 octave increments; 25 ms duration; 5 ms ramps) at eight sound intensities (0–70 dB SPL in 10 dB increments) at a rate of one tone per second.

### 2.3. Electrophysiological Data Analysis

The characteristic frequency (CF) of a cortical site was defined as the frequency at the tip of the V-shaped tuning curve. For flat-peaked tuning curves, the CF was defined as the midpoint of the plateau at threshold. For tuning curves with multiple peaks, the CF was defined as the frequency at the most sensitive tip (i.e., with lowest threshold). Response bandwidths 20 dB above the threshold of tuning curves (BW20) were measured for all sites. The CF, threshold, and BW20 were determined using an automated routine developed in the MATLAB environment (The MathWorks Inc., Natick, MA). Primary auditory cortex (A1) was identified based on its rostral-to-caudal tonotopy, reliable short-latency tone-evoked neuronal responses, and relatively sharp V-shaped RF [33].

To generate A1 maps, Voronoi tessellation (a MATLAB routine; The MathWorks Inc.) was performed to create tessellated polygons with electrode penetration sites at their centers. Each polygon was assigned the characteristics (i.e., CF) of the corresponding penetration site. In this way, every point on the surface of the auditory cortex was linked to the characteristics experimentally derived from its closest sampled cortical site. The boundaries of the primary auditory cortex were functionally determined using the following criteria: (1) primary auditory neurons generally have a continuous, single-peaked, V-shaped receptive field and (2) CFs of the A1 neurons are tonotopically organized with high frequencies represented rostrally and low frequencies represented caudally [34].

To test how the mean firing rates of each neuron were modulated by the target and nontarget test stimuli, signal-detection theory was applied to generate receiver operating characteristic (ROC) curves [35]. For each A1, two distributions of average neuronal firing rates were constructed. One distribution contained the average firing rate from each A1 neuron during the presentation of the target stimulus and the other contained the same information but for the nontarget stimulus. From these two distributions, an ROC curve was generated. The area under this curve represented the probability that an ideal observer could differentiate between the two distributions [36]. An ROC value of 0.5 indicates that the two distributions overlap completely and that an ideal observer can only differentiate between these distributions by chance. An ROC value of 1.0 indicates that the two distributions do not overlap and that an ideal observer can perfectly differentiate between the firing rates elicited by the target and nontarget stimulus.

### 2.4. Immunohistochemistry

Following electrophysiological recordings, all rats received a high dose of ketamine/xylazine/acepromazine (130/26/3 mg/kg, i.p.) and were perfused intracardially with phosphate buffered saline (pH 7.4, PBS) followed by paraformaldehyde (4%) in 0.1 M PBS. Their brains were removed from the skulls, postfixed in the same fixative overnight, transferred to a 30% sucrose solution, snap-frozen, and stored at −80°C until sectioning. Fixed material was sectioned on a freezing microtome at a 40 *μ*m thickness in the coronal plane along the tonotopic axis of A1. The cortical borders were defined according to the cell size, density, and depth as in [37]: layer I (0–175 *μ*m), layers II-III (175–500 *μ*m), layer IV (500–700 *μ*m), and layers V-VI (700–1200 *μ*m).

Brain slices were treated with PBS 0.1 M 3 × 5 min followed by a mixture of gelatine (2%) and triton X-100 (0.25%) in PBS (PBS-GT) for 4 × 10 min, transferred into primary antibody solution containing PBS-GT, and incubated overnight. After incubation, the sections were washed in blocking buffer PBS-GT and incubated for one hour in dilutions of secondary antibody conjugated with different fluorophores. All primary and secondary antibodies used (see below) were tested for optimal conditions for single and double labeling. We used the following antibodies to label the brain tissue: (1) rabbit anti-SOM (Peninsula Laboratories #T-4103, 1 : 2000), (2) goat anti-ChAT (Chemicon #AB144P, 1 : 200), (3) donkey anti-goat (conjugated to Alexa Fluor (AF647), 1 : 800, Jackson ImmunoResearch, West Grove, PA), and (4) donkey anti-rabbit (AF488, 1 : 800, Jackson). Stained sections were mounted on 1% gelatin-coated slides, air-dried, and cover-slipped with Mowiol solution (Tris 0.2M, 30% glycerol, and 12% Mowiol). Brain tissue was immunostained in pairs to limit variability related to antibody fixation, incubation time, and postsectioning condition of tissues.

### 2.5. Microscopy, Image Acquisition, and Data Analysis

A Zeiss LSM 510 Meta confocal microscope equipped with filter for green Cy2/AF488, red CY3, and infrared CY5/AF647 was used to assess fluorescence in the immunostained sections. To locate A1 in nonfunctionally mapped animals, we used the stereotaxic coordinates (Paxinos): interaural between 5.76 and 2.16 mm and Bregma between −3.24 and −6.84 mm (see the above section on determination of A1 borders). To quantify the positive cells, 21 digital images of A1 cortical sections were taken with a 40x objective (Zeiss LSM 510) at random locations within each A1 of each hemisphere for each animal. All quantifications were assessed in 400–500 *μ*m wide A1 sectors (the approximate width of A1 on coronal sections) per hemisphere extending from layer 1 to the underlying white matter. Confocal images were thresholded and adjusted for brightness to maximize the dynamic range of each channel using ImageJ (http://rsb.info.nih.gov/ij/) and Adobe Photoshop CS5 (Adobe, San Jose, CA).

We determined the number of immune-labeled cells in each section of A1 using the optical dissector method (Stereo Investigator software, MBF Bioscience, Williston, VT) to avoid biased sampling. These counts were then pooled and adjusted to reflect what would have been counted in the whole 40x field. Data were then recorded as an averaged value per high power field (hpf) for each animal and group. All cells displaying labeling above background levels were counted, regardless of their staining intensity. Data from both hemispheres was pooled. An observer blind to the group membership of the animal performed all cell counts.

Unless specified otherwise, statistical significance was assessed using unpaired two-tailed *t*-tests. Data are presented as mean ± standard error (SE).

## 3. Results

### 3.1. Reduction in Choline Acetyl Transferase (ChAT) and Gamma Activity in the Older A1

**Figure 1:**
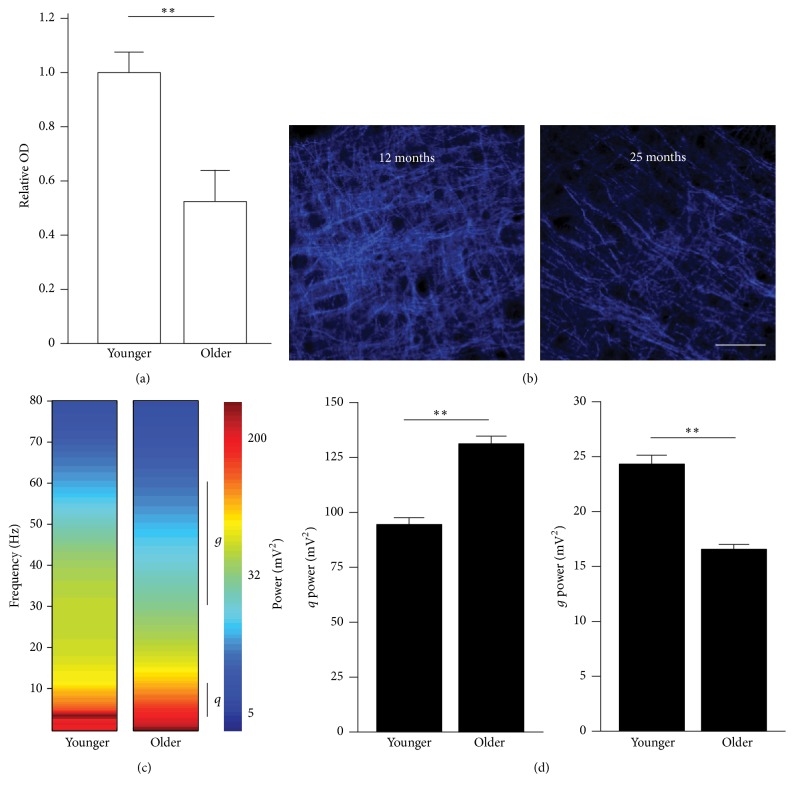
Reduction in Choline Acetyl Transferase (ChAT) and gamma activity in the older A1. (a) Average density of ChAT staining in young and older A1 determined by immunohistofluorescence (primary Ab: anti-ChAT; secondary Ab: AF647). (b) High power (40x) photomicrograph showing staining for ChAT in layer 4 of A1 in one younger (12-month-old) and one older (25-month-old) rat. (c) Power spectral density of local field potential (LFP) signals recorded during the presentation of tone pips in younger and older rats. The theta (*θ*) and gamma (*γ*) range is shown by vertical black bars. (d) Average theta (left) and gamma (right) band power in the LFP signals recorded in younger and older A1 during pure tone presentation. Note the relative increase in theta and decrease in gamma consistent with loss in cholinergic tone in A1 (younger: *n* = 6, recorded sites for LFP = 586, hemispheres examined for ChAT staining, *n* = 8, and number of photomicrographs analyzed, *n* = 24; older: *n* = 5, recorded sites for LFP = 476, hemispheres examined for ChAT staining, *n* = 8, and number of photomicrographs analyzed, *n* = 24). Scale bar: 50 *μ*m. Values shown are mean ± SE. ^*∗*^ *p* < 0.05: ^*∗∗*^ *p* < 0.01: *t*-test.

To first confirm the effect of aging on the cholinergic system, we compared the density of Choline Acetyl Transferase (ChAT) staining obtained from both young and old naïve untrained rats (see Figure 1). We found that the ChAT density was significantly reduced in older rats compared to young ones (*t* = 3.23, *p* = 0.002), consistent with the finding of degenerating cholinergic cells in the basal forebrain of the aging brain [15, 16] and in afferent cortical projections [17, 18]. We next investigated the effect of aging on a correlate of cholinergic activity: the gamma power obtained from local field potential (LFP) signals during the presentation of tone pips of various frequencies and intensities. In good agreement with the previous result, we found a significant reduction in gamma (*γ*) power (30–60 Hz) in older rats (*t* = 9.30, *p* < 0.001) that was accompanied by a significant increase in theta (*θ*) power (3–12 Hz) (*t* = 2.60, *p* = 0.009; also see Figure 1). This is consistent with previous reports showing that an increase of cholinergic activity is associated with a decrease of theta power and an increase in gamma power within rat auditory cortex [38, 39]. Overall, we found that aging is associated with a reduction in ChAT density and with an increase in the theta/gamma power ratio within auditory cortex.

### 3.2. Impact of Training and Rivastigmine on Discrimination Learning in Young and Old Rats

**Figure 2:**
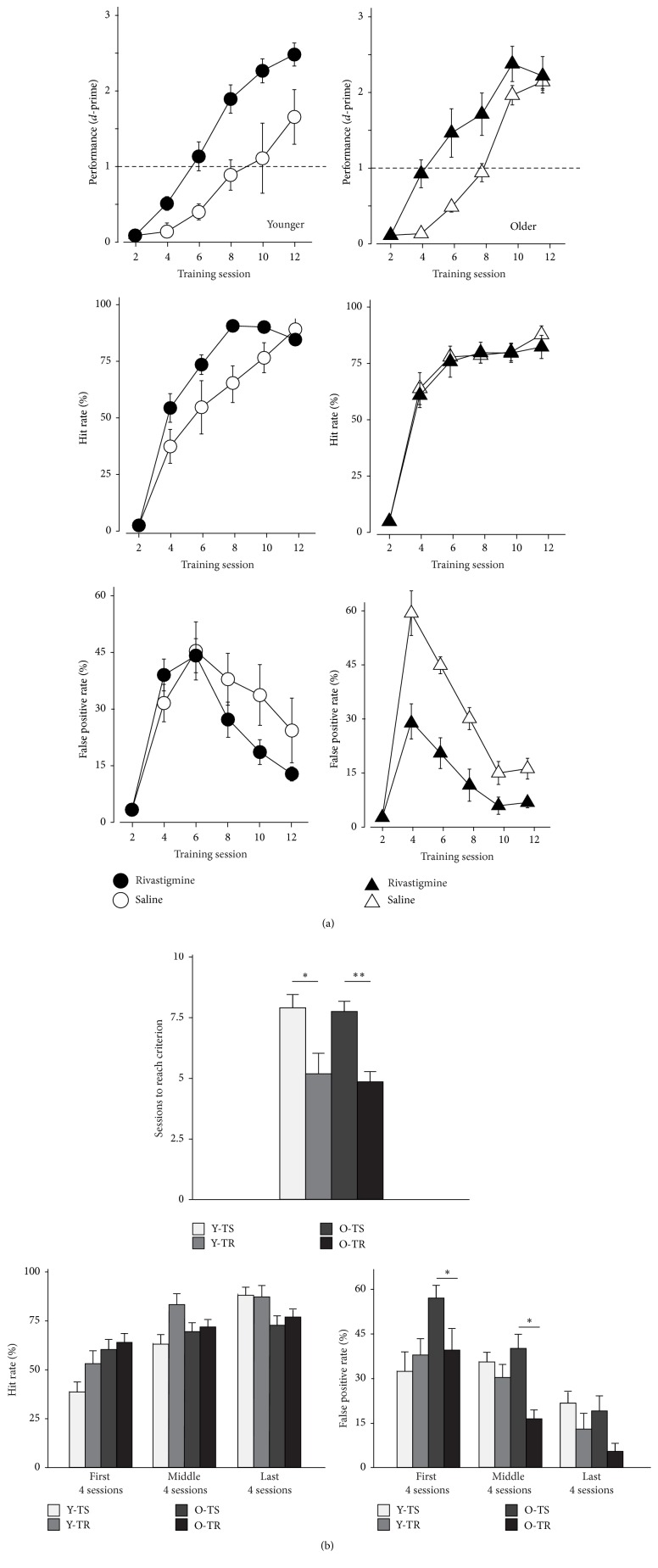
Impact of rivastigmine on auditory discrimination learning in young and older rats. Young (12–14 months old, *n* = 11) and aging (24–30 months old, *n* = 9) rats were trained on a two-tone discrimination task. In this go-no-go experimental paradigm, rats were rewarded with food for performing a behavioral response only when a 5 kHz tone was presented. The nontarget was always a 10 kHz tone. Lack of response to a target (miss) or a response to a nontarget (false positive) resulted in a delay before the next trial. One group of younger (Y-TR, *n* = 6) and older rats (O-TR, *n* = 4) was administered rivastigmine orally prior to each training session. The other younger and older groups (Y-TS, *n* = 5, and O-TS, *n* = 5) were administered an equivalent volume of saline before training. (a)* Top row*: average performance of younger and older rats on the training across time. A* d*-prime of 1 was used as the main criterion to determine mastery on the task.* Middle row*: average hit rate for each experimental group as a function of training sessions completed.* Bottom row*: average false positive rate for each experimental group as a function of training sessions completed. (b)* Top row*: average number of sessions to reach criterion in all experimental groups.* Bottom rows*: average hit rate and false positive rate, respectively, for the first and last four training sessions in all experimental groups. Values shown are mean ± SE. ^*∗*^ *p* < 0.05: ^*∗∗*^ *p* < 0.001: *t*-test.

The performance of both young and old rats improved steadily over 9 to 12 one-hour sessions (see Figure 2). The administration of the cholinesterase inhibitor rivastigmine tartrate had a significant effect on the learning rates of both young (*t* = 7.04, *p* = 0.03) and old rats (*t* = 10.61, *p* = 0.01) by reducing the amount of sessions required to reach a criterion of* d*-prime >1, usually considered a marker of successful discrimination between target and nontarget stimuli. While both age groups showed overall improvement in the task, the specific manner in which they did so differed. Cholinergic enhancement in the young rats led to a significant increase in the hit rate (HR) (*t* = 4.91, *p* = 0.05) without affecting the average false positive rate (FPR) (*t* = 1.04, *p* = 0.34). In marked contrast, cholinergic enhancement in the older rats had the opposite effect where the FPR was significantly reduced compared to saline treated animals (*t* = 16.70, *p* = 0.005) without affecting the average hit ratio (*t* = 0.39, *p* = 0.55). The effect of rivastigmine on the FPR in older rats seemed to be stronger at the onset of training and during the initial learning phase more so than once the task was learned, as evidenced by the significant difference between groups for the first four sessions pooled together (*p* = 0.03) and for the middle four sessions (*p* < 0.001), and by the absence of a significant difference for the last four sessions (*p* = 0.14). To summarize, rivastigmine improved the learning rates in both age groups, but it did so in different manners for each group. In the young group, it improved the detection of the target stimulus, whereas it reduced responses to the nontarget in the old group.

### 3.3. Impact of Training and Rivastigmine on A1 Frequency Tuning

**Figure 3:**
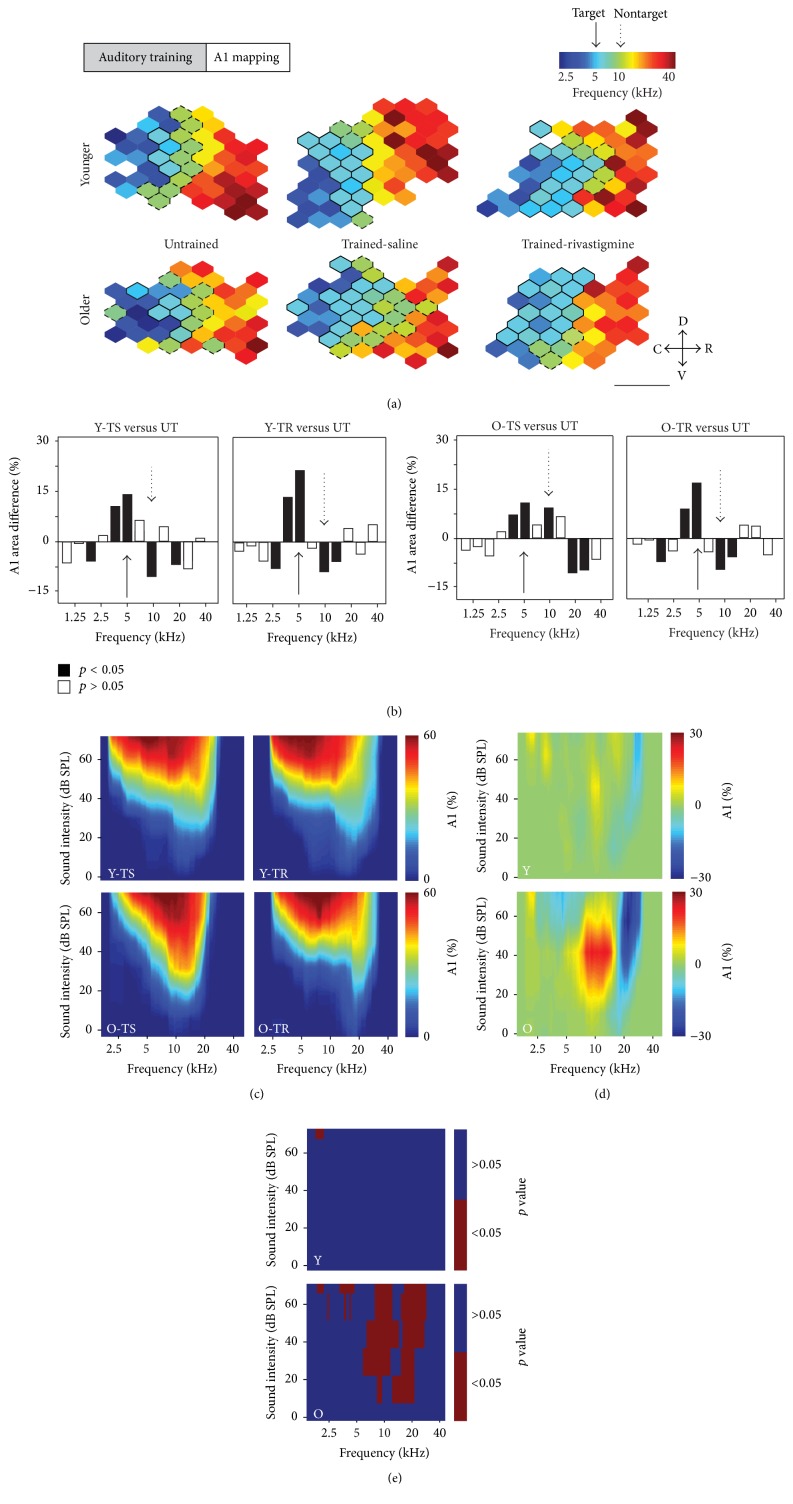
Impact of training and rivastigmine on A1 frequency tuning. (a) Representative A1 characteristic frequency (CF) maps from naïve (untrained), trained with saline (TS), and trained with rivastigmine (TR) young and older rats. Bolded polygons have a CF at the target tone ±0.3 octaves. Hatched polygons have a CF at the nontarget tone ±0.3 octaves. Note the increase in map area to the nontarget tone in the O-TS group only. (b) Difference in A1 area tuned to various frequencies between each experimental group and untrained animals. The full arrow points to the target frequency; the hatched arrow points to the nontarget frequency. Note how in each group except O-TS there is a significant reduction in area tuned to the nontarget frequency. (c) Percentage of A1 activated by every frequency-intensity combination used for mapping in all experimental groups. (d) Difference in the percent of activation between Y-TS and Y-TR (*top*) and O-TS and O-TR (*bottom*). (e) Plot showing statistically significant difference in A1 activation in the young (top) and older (bottom) groups. Scale bar represents 1 mm. D: dorsal; C: caudal; R: rostral; V: ventral (Y-UT: *n* = 8, recorded sites = 435; Y-TS: *n* = 5, recorded sites = 257; Y-TR: *n* = 6, recorded sites = 312; O-UT: *n* = 8, recorded sites = 412; O-TS: *n* = 5, recorded sites = 261; O-TR: *n* = 5, recorded sites = 249). Values shown are mean ± SE. ^*∗*^ *p* < 0.05: *t*-test.

Rats in the experimental groups were all trained to discriminate between a target tone (5 kHz) and a nontarget (10 kHz) tone. To examine the effects of training and cholinergic enhancement on the cortical representation of each frequency, we first compared the number of A1 neurons whose characteristic frequency (CF) was within ±0.3 octaves of either the target or the nontarget frequency (see Figure 3(a)). Compared to young untrained rats, both trained young groups showed an increase in the number of neurons with a target CF (Y-TS (trained with saline): 16.4% increase in the proportion of neurons responding to the tone, *p* = 0.004; Y-TR (trained with rivastigmine): 21.7% increase, *p* = 0.003) while showing a decrease in the number of neurons with the nontarget CF (Y-TS (10.6% decrease, *p* = 0.02), Y-TR (8.4% decrease, *p* = 0.04)). Both old trained groups showed an increase in the number of neurons with a target CF compared to the untrained group (O-TS (12.7% increase, *p* = 0.02), O-TR (18.8% increase, *p* = 0.01)). However, while the O-TR group showed a decrease in the number of neurons with a nontarget CF (9.7% decrease, *p* = 0.02), the O-TS group showed an increase (10.5% increase, *p* = 0.04).

We next investigated the percentage of A1 that was activated by every frequency-intensity combination used for mapping (see Figures 3(c)–3(e)). When directly comparing the rivastigmine and saline old groups, we observed a significant increase in the percentage of A1 responding to frequencies between 8.04 and 16.2 kHz for sound intensities between 10 and 70 dB SPL (8% to 27% difference, 0.04 < *p* < 0.008, with Bonferroni correction) combined with a significant decrease for frequencies between 18.6 and 30.3 kHz for sound intensities between 10 and 70 dB SPL (10% to 30% difference, 0.03 < *p* < 0.01, with Bonferroni correction). The same comparison in the young only revealed a small reduction in the percentage of neurons responding to frequencies between 1.6 and 2.1 kHz at a sound intensity of 70 dB for the rivastigmine group (6% decrease, *p* = 0.04, with Bonferroni correction). Overall, training alone increased the ratio of neurons having a CF corresponding to the target frequency compared to the nontarget frequency in the young rats, whereas it only increased the neural representation of the target frequency in the older rats. The administration of rivastigmine was sufficient to reduce the neural representation of the nontarget frequency in the old rats.

### 3.4. Training and Rivastigmine Effects on Cortical Auditory Responses to the Training Tones

**Figure 4:**
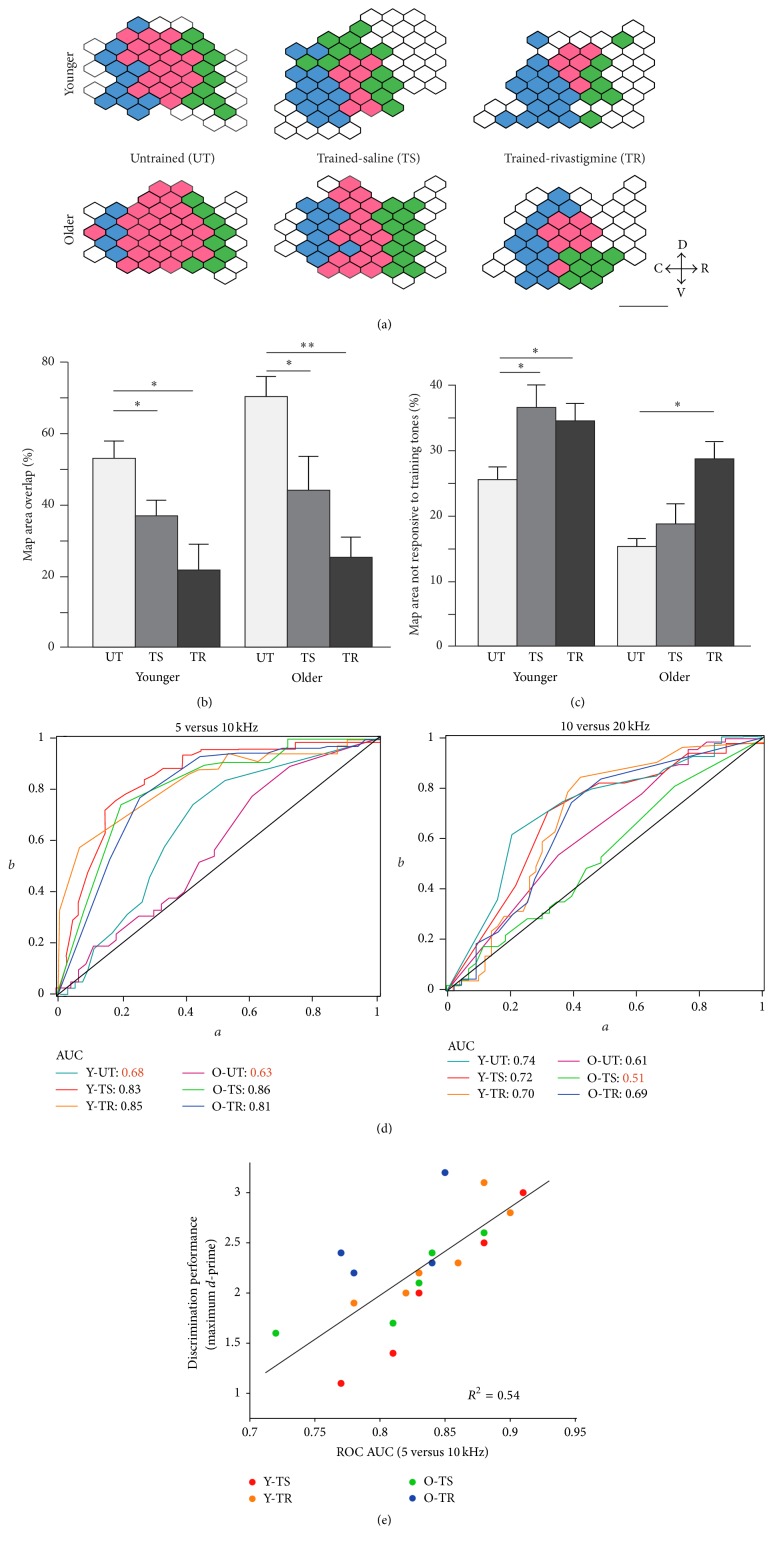
Training and rivastigmine impact on the overlap in A1 area responsiveness to training tones. (a) A1 maps from the same animals as in Figure 3 showing the area activated by 5 kHz (blue polygons) or 10 kHz (green polygons) tones presented at 60 dB SPL (the sound intensity during training). The dark pink polygons indicate the area of the map activated by both frequencies. (b) Difference in A1 area overlap in responsiveness to 5 and 10 kHz ± 0.3 octaves in all groups. Note how training is associated with an overall reduction in overlap in all groups. (c) Difference in A1 area responsiveness to frequencies other than 5 or 10 kHz in all groups. Note how training resulted in a relative increase of A1 area activated by nontrained tones in all groups except O-TS where the reduction in overlap (b) was driven by a relative expansion of the area responsive to 10 kHz. (d)* Left*: ROC analysis demonstrating the average discriminability in the pattern of A1 activation for 5 and 10 kHz tones presented at 60 dB SPL.* Right*: ROC analysis demonstrating the average discriminability in the pattern of A1 activation for 10 and 20 kHz tones presented at 60 dB SPL. Note how training decreases the AUC in the O-TS group only. (e) Maximal performance (*d*-prime) on behavioral training plotted against AUC (5 versus 10 kHz) for all groups. Scale bar represents 1 mm. D: dorsal; C: caudal; R: rostral; V: ventral (Y-UT: *n* = 8, recorded sites = 435; Y-TS: *n* = 5, recorded sites = 257; Y-TR: *n* = 6, recorded sites = 312; O-UT: *n* = 8, recorded sites = 412; O-TS: *n* = 5, recorded sites = 261; O-TR: *n* = 5, recorded sites = 249). Values shown are mean ± SE. ^*∗*^ *p* < 0.05: ^*∗∗*^ *p* < 0.001: *t*-test.

We next investigated the proportion of A1 that responded to 60 dB tones for either the target frequency, the nontarget frequency, or both of them, regardless of the CF. Figures 4(a)–4(c) illustrate the overlap in A1 area that was responsive to both tones and how training and the administration of rivastigmine tended to reduce the overlap area and increase the area that responded to neither of the training tones. In the young, both training alone (*p* = 0.04) and training with rivastigmine (*p* = 0.008) significantly reduced the area of overlap compared to untrained animals, while significantly increasing the map area not responsive to either of the training tones (Y-TS: *p* = 0.05; Y-TR: *p* = 0.05). The reduction of overlap area was equally observed in the older groups (O-TS: 0.04; O-TR: *p* < 0.001), whereas the increase in area not responsive to training tones was only significant in the rivastigmine group (O-TS: *p* > 0.2; O-TR: *p* = 0.02).

To further investigate the ability of A1 to discriminate between both tones, we performed receiver operating characteristic (ROC) analyses that allowed us to characterize the performance of a binary classifier system. More precisely, the area under the ROC curve quantifies the overall ability of A1 to discriminate between both tones (presented at 60 dB). Compared to the untrained groups (see Figure 4(d)), all trained groups showed an increase in the area under the ROC curve (Y-UT versus Y-TS: *p* = 0.05, Y-UT versus Y-TR: *p* = 0.04, O-UT versus O-TS: *p* = 0.004, and O-UT versus O-TR: *p* = 0.05; ANOVA with post hoc Tukey tests) that corresponded with enhanced discriminability between the training tones. No differences were found between old and young rats of the same training/ACh condition. In contrast, when comparing the discriminability between the nontarget tone and an untrained tone (10 kHz and 20 kHz), training caused a reduction in the area under the curve for the O-TS group compared to the O-TR, Y-TR, Y-TS, and Y-UT groups (all *p* > 0.05) and also for the O-UT group compared to the Y-UT group (*p* > 0.05), suggesting that for all other trained groups the training did not alter the ability of A1 to discriminate between the nontarget frequency and a distinct untrained frequency.

Finally, to relate the behavioral performance of the trained rats with A1's ability to discriminate between the target and the nontarget, we correlated the individual ROC area under the curve values (using the 5 and 10 kHz tones) with the maximal performance achieved by each animal (maximal* d*-prime value measured over the course of the training). When all groups were pooled together, the area under the ROC curve explained 54% of the variance found in the maximal performance reached by the trained animals (see Figure 4(e)). Importantly, we found that the relationship between both variables appeared to be consistent across all groups (i.e., all followed a similar trend line) and that, regardless of group membership, the better the discriminability of A1 neurons' firing rates, the better the behavioral performance.

### 3.5. Changes in Tuning Bandwidth and Threshold Subsequent to Training

**Figure 5:**
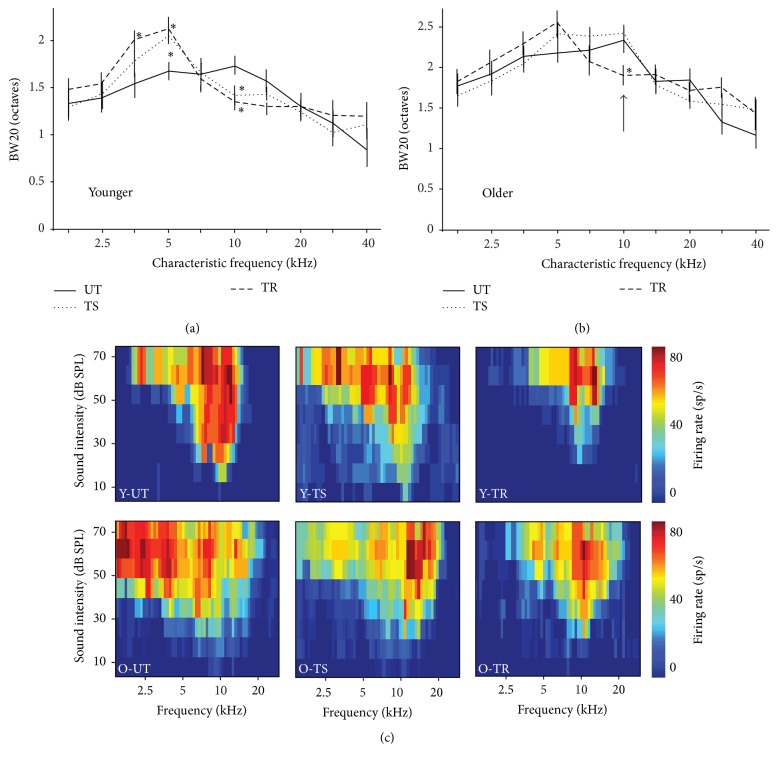
Changes in tuning bandwidth subsequent to training in the different experimental groups. ((a)-(b)) Average BW20 for all neurons recorded in young and older untrained (UT), trained with saline (TS), and trained with rivastigmine (TR) groups and separated by CF. Representative receptive fields of A1 neurons in the same experimental groups. The black arrows point to the lack of change in BW20 in the O-TS group compared to all other groups. (c) Representative tuning curves from each group for neurons with a CF of the nontarget tone (10 kHz) that illustrate the narrowing of the bandwidth in all groups except the O-TS group (Y-UT: *n* = 8, recorded neurons = 189; Y-TS: *n* = 5, recorded neurons = 132; Y-TR: *n* = 6, recorded neurons = 157; O-UT: *n* = 8, recorded neurons = 201; O-TS: *n* = 5, recorded neurons = 138; O-TR: *n* = 5, recorded neurons = 117). Values shown are mean ± SE. ^*∗*^ *p* < 0.05: *t*-test.

In addition to changes at the level of the tonotopic map (in terms of both CF and frequency-response patterns at 60 dB), training was found to have significant effects on the tuning bandwidths of A1 neurons (by comparing the response bandwidth at 20 dB above threshold (BW20); see Figure 5). In young rats, training both with and without rivastigmine led to a widening of the tuning bandwidth of neurons with a CF corresponding to the target frequency (Y-TS: *p* = 0.02; Y-TR: *p* = 0.03) in combination with a narrowing of the tuning bandwidth for the nontarget frequency (Y-TS: *p* = 0.03; Y-TR: *p* = 0.02). In old rats, however, training was not sufficient to significantly alter the tuning bandwidth of neurons with a CF corresponding to the target frequency (O-TS: *p* = 0.26; O-TR: *p* = 0.1), whereas only training with rivastigmine narrowed the tuning bandwidth of neurons with a CF corresponding to the nontarget (O-TS: *p* = 0.69; O-TR: *p* = 0.03). Finally, both training and the administration of rivastigmine did not have an effect on the auditory thresholds required to evoke a cortical response in either the young or the aged rats. It should also be noted that there was no significant difference in cortical thresholds between younger and older groups (*p* > 0.2).

### 3.6. Effect of Training and Rivastigmine on the Number of SOM+ Cortical Interneurons

**Figure 6:**
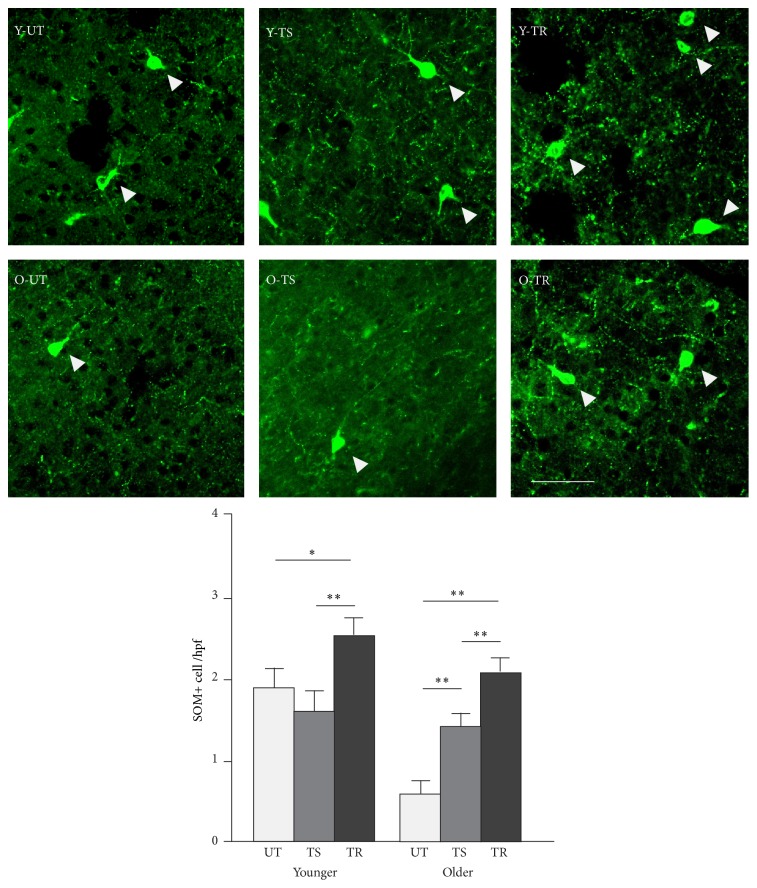
Effect of training and rivastigmine on the number of SOM+ cortical interneurons. Quantitative analysis of the average number in A1 of SOM immunoreactive cells per high power field (hpf) in all experimental groups. Top panel: representative high power photomicrographs of representative sections in all groups. Bottom panel: average SOM+ cell counts in all groups (all layers pooled). Number of hemispheres examined: Y-UT = 10, Y-TR = 8; O-UT = 8, O-TR = 8; number of micrographs/hemispheres: 7. ^*∗*^ *p* < 0.05, ^*∗∗*^ *p* < 0.01, *t*-test. Scale bar: 50 *μ*m.

Somatostatin positive (SOM+) cells, a class of GABAergic interneurons, are the primary target of cortical cholinergic projections and play an important role in the neuromodulation of sensory processing and learning [40–43]. For this reason, we performed quantitative analysis of the average number of SOM immunoreactive cells per A1 high power field (hpf) performed for all experimental groups (see Figure 6). In the young, while training alone did not have an effect on SOM+ cell count (*p* = 0.1), training with rivastigmine significantly increased the number of SOM+ cells compared to the untrained group (*p* = 0.01) and the Y-TS group (*p* = 0.005). In the old rats, not only did both trained groups show an increase in the number of SOM+ cells (O-TS: *p* < 0.001; O-TR: *p* < 0.001), but also those having received rivastigmine had an even greater number of SOM+ cells than the other trained group (*p* = 0.006).

## 4. Discussion

The purpose of the present study was to investigate the effect of a cholinesterase inhibitor (rivastigmine tartrate) on both brain function and behavior and how these effects might differ in young and old rats given the important cholinergic deficit observed in older rats (see Figure 1). While it is clearly established that cholinergic enhancement boosts perceptual learning in young adults, little is known about whether boosting a deficient cholinergic system in the elderly would help reduce the gap that exists between young and aged adults in terms of their perceptual learning rates. Furthermore, we also wanted to document how cholinergic enhancement differentially affects the expression of auditory cortical plasticity mechanisms associated with perceptual learning in both young and older adults.

Behaviorally, the administration of rivastigmine 30 minutes prior to each training session significantly improved the performance of both young and old adult rats compared to control groups of the same age who were only given a saline solution (see Figure 2). However, the means by which both age groups improved differed. In the young rats, boosting the cholinergic system significantly improved their overall hit rate (correct detection of target stimulus), which led to better discrimination performance. In the old rats, while cholinergic enhancement had little effect on the hit rate, it significantly reduced the false positive rate (incorrectly responding to the nontarget stimulus), which led to a similar improvement in discrimination performance to that observed in the young adults. This reduction was particularly evident during the middle four training sessions when those having received rivastigmine showed a significant jump in performance (as indicated by the* d*-prime measure).

Aging is associated with deficits in the ability to suppress task-irrelevant distracting information in combination with the inability to sustain focus on goal-relevant target information, which disrupts the successful accomplishment of task-relevant goals [44, 45]. While previous work has shown that operant behavioral auditory training paradigms significantly reduce the false positive rate in aged adult rats [29], the present findings indicate that directly acting on the cholinergic system further accentuates the drop in false positives. The increase in performance in both young and aged rats further supports previous findings that have shown that ACh can improve stimulus discrimination [13, 46, 47], whereas the finding of a reduction of false positives in the aged rats supports the literature demonstrating the fundamental role played by ACh in attentional mechanisms of cognitive control [48, 49].

### 4.1. Cholinergic and Training-Induced Changes in Primary Auditory Cortex

Both behavioral training and the daily administration of rivastigmine were found to have profound effects on auditory cortex plasticity. Behavioral training alone led to an overrepresentation of the target tone and an underrepresentation of the nontarget tone in the primary auditory cortex of young adult rats, whereas it led to an overrepresentation of both tones in the aged rat (see Figure 3). The addition of rivastigmine produced similar effects to training alone in the young rats (while producing a 5.3% increase of the representation of the target tone compared to the training alone), whereas it significantly reduced the representation of the nontarget tone in A1 of the aged rats compared to both the untrained and the trained A1. The increase in the representation of a behaviorally relevant auditory target stimulus is a plasticity mechanism that is directly linked to perceptual learning and is consistent with several previous reports [6, 33, 50]. The further increase in representation in the young rats following cholinergic enhancement is, at least in part, likely responsible for their significant increase in the correct detection of the target stimuli. The underrepresentation of the nontarget is consistent with previous reports [51] and likely constitutes a processing strategy that aids in ignoring the nonrelevant stimuli. Moreover, the fact that behavioral training alone was not sufficient to produce this underrepresentation in the elderly rats suggests that the cholinergic system is necessary for the development of this specific plasticity mechanism within A1 and further supports the notion that the cholinergic system plays a key role in our ability to inhibit the processing of and ignore nonrelevant stimuli [52–54]. Furthermore, not only did training alone in the old rats prevent an underrepresentation of the nontarget tone, but also it in fact led to an overrepresentation of it. This overrepresentation likely explains the higher false positive rate observed in this group and is consistent with previous findings showing that while behavioral training in old rats improves many aspects of auditory processing, it has limited success in improving distractor processing [29].

Auditory training and cholinergic enhancement also had a significant effect on two other measures of auditory cortical processing. The first relates to the area size of A1 that responds to both the target and the nontarget tone when presented at a moderately high intensity (60 dB). In untrained rats, slightly more than half of A1 was responsive to both tones at 60 dB in the young whereas the same could be said for just over two-thirds of A1 in the older rats. However, auditory training produced a dramatic reduction in the overlapping area (by 30% in the young and 36% in the old), and this reduction was further increased by the administration of rivastigmine (by another 30% in the young and 37% in the old). In the younger groups, this reduction in overlap area was also accompanied by a significant increase in the map area not responding to both training tones likely due to the reduction in A1 area responsive to the nontarget. This effect was not observed in the older training group that received saline in which only a relatively small fraction (18%) of A1 remained responsive to nontraining tones after training. The combination of training and cholinergic enhancement in the end reduced the overlapping area (i.e., that is responsive to both tones) to 26% in the old rats and to 21% in the young rats. This suggests that when combined with operant training paradigms, the administration of rivastigmine leads to substantial plastic changes within A1 where the areas that are responsive to either tone become better segregated. In turn, this better segregation is likely to lead to a better discriminability of the two training tones and other irrelevant nontrained tones by A1. This hypothesis was further confirmed by performing ROC discriminant analyses that estimated the average ability of A1 neurons to properly discriminate between both trained tones and a trained tone and one irrelevant tone (10 versus 20 kHz) (see Figure 4). Briefly, the ROC curve is a graphical plot that illustrates the performance of a binary classifier system and the area under the ROC curve (AUC), in this specific instance, represents the average probability that A1 as a whole will be able to discriminate between both tones [36]. Here, we showed that the AUC associated with both training alone and in combination with rivastigmine was significantly increased compared to control groups for both old and young rats. Interestingly, no difference was observed between old and young rats of the same training/ACh condition, suggesting that cholinergic enhancement did not add much to the average ability of A1 neurons to discriminate between the target and nontarget for both young and old adult rats. Furthermore, when pooling the ROC data from all groups together, we show that the AUC is a great predictor of the behavioral performance of both old and young rats and explains 56% of the variance seen in the performance level reached by each rat. In other words, there is a good correspondence between the average A1 neuronal firing rate patterns in response to the two tones and the performance of the animal following auditory discrimination training.

The other measure that was significantly modulated by training and cholinergic enhancement is the frequency tuning bandwidth of neurons, which is generally considered to be a good measure of frequency selectivity of A1 neurons (i.e., the degree to which a neuron responds to frequencies other than its CF). Training alone was sufficient to drive bandwidth changes for neurons tuned to either the target or the nontarget tone in the young; the addition of rivastigmine did not lead to any further changes. Auditory training caused a widening of the bandwidth for neurons tuned to the target tone, whereas it led to a narrowing of the bandwidth for neurons tuned to the nontarget. These tuning changes likely occurred to enhance the detection of tones near the target frequency and to reduce neuronal responses to the nontarget frequency. A similar widening of the tuning bandwidth for neurons tuned to the target frequency was observed in the old rats (though the effect did not reach statistical significance). However, the administration of rivastigmine was necessary to narrow the bandwidth of neurons tuned to the nontarget in the old rats. This is consistent with other above-highlighted measures of auditory processing of the nontarget, in that cholinergic enhancement is necessary to induce cortical changes that increase the ability of old rats to ignore nontarget stimuli. Finally, although the bandwidth for neurons tuned to the target frequency was similar between young and old rats (see Figure 5), it was substantially higher for neurons tuned to the nontarget in the older rats, consistent with previous reports showing that, in general, A1 neurons are more broadly tuned in older rats [29, 55].

The finding of broadened tuning bandwidths in A1 neurons tuned to the target tone was partially surprising given that auditory training on frequency discrimination tasks usually leads to a narrowing of the tuning bandwidth, thereby increasing the frequency selectivity of auditory neurons [29, 56]. Indeed, broader tuning curves lead to wider stimulus-induced cortical activation, making sensory discrimination more based on spatial activation of the cortex and therefore generally less reliable [6, 57]. However, the task used here was a two-tone frequency discrimination that is relatively easy to perform compared to previous adaptive staircase procedures that are geared towards improving perceptual resolution [29, 56]. Indeed, here rats need not develop better frequency resolution to be positively reinforced for the present task; they simply need to learn to recognize one tone and to ignore the other. Consequently, discrimination based on spatial activation of A1 is therefore likely appropriate in this specific training context, especially given the additional finding of a decreased spatial overlap of A1 areas that are responsive to the target and nontarget tone following training. This coding strategy, however, is likely somewhat unique and specific to the type of discrimination that was used and would not be efficient in a different context where, for example, the target and nontarget would have varied between training sessions.

Similarly, the bandwidth effects observed here are likely to be highly dependent on the type of task/training performed and are not generalizable to all contexts. As highlighted above, an overall reduction in bandwidth across all frequencies in A1 was observed for training paradigms that involved roving nontargets [29] or combined roving targets and nontargets [56]. Consequently, the plastic tuning changes in A1 that result from behavioral training seem to be tightly linked to the relevant sensory information required to perform the task.

Lastly, behavioral training and cholinergic enhancement also led to marked structural changes within A1. While both caused a significant increase in the number of SOM+ cells in the old rats, only the addition of rivastigmine led to an increase in SOM+ numbers in the young. SOM+ cells are a class of inhibitory interneurons that, among other functions, play a key role in the neuromodulation of sensory processing and learning [41–43]. They are also the main target of cholinergic projections in the cerebral cortex [40]. Within auditory cortex, SOM+ cells have been shown to decline in number with advancing age [58]. However, here we show that both auditory training and cholinergic enhancement during auditory training can rescue this decline in numbers observed in old rats. The relationship between behavioral training and somatostatin has been scarcely investigated, though there is some evidence that sensory training in the tactile modality in mice can increase the number of SOM+ cells within somatosensory cortex [59] and that boosting somatostatin levels can improve learning and memory [60]. Interestingly, the latter finding was only observed in aged mice, and not younger ones, consistent with our own findings. Finally, how boosting the cholinergic system directly affects the number of SOM+ cells within auditory cortex remains unclear to this point. While we do know that somatostatin cells contain cholinergic and muscarinic receptors [40] and can be depolarized by cholinergic agonists [61–63], further studies should aim to identify whether a clear causal link exists between increased cholinergic activity and somatostatin cell density.

In conclusion, we show here the powerful potentiating effect of acetylcholine on perceptual learning in both young and old adult rats. Cholinergic enhancement was shown to accelerate the learning rate for discrimination between target and nontarget tone in both age groups, although this perceptual benefit was achieved in a different manner by each group. The benefit in young rats was achieved by increasing the correct detection of the target, whereas it was achieved by reducing the incorrect responses to the nontarget in the older rats. The latter finding is consistent with the notion that acetylcholine is an effective agent for reducing distractibility in older individuals. Cholinergic enhancement also had significant plastic changes on auditory cortical processing mechanisms within A1 when compared with behavioral training alone, particularly in the older group. In general, the combination of auditory training and cholinergic enhancement was found to restore many cortical processing features that are typical of the young brain, which highlights the great potential that combining behavioral and cognitive training with cholinergic neuromodulation has in recovering or preventing age-related cognitive and sensory deficits.
